# 

**Published:** 1882-05

**Authors:** 


					﻿ESTABLISHED IN 1855.
OldSerie’.	MAV 1RR9	New Series
Vol. XX—No. 2.	111/IX, 1004.	Vol. 1-No. 8.
ATLANTA
MEDICAL REGISTER.
(ATLANTA MEDICAL AND SURGICAL JOURNAL.)
ZDITOES:
JNO. THAD. JOHNSON, M.D., JAMES B. BAIRD, M.D.
PUBLISHED BY H. H. DICKSON, AT $2.50 A YEAR IN ADVANCE
ATLANTA, GEORGIA:
II. II. DICKSON, BOOK, AND JOB PRINTER AND BOOKBINDER.
1882.
				

